# Microbial community of civet excreta fed by robusta cherry coffee in Indonesian civet coffee production

**DOI:** 10.5455/javar.2025.l921

**Published:** 2025-06-02

**Authors:** Sri Winarsih, Uswatun Hasanah, Lilis Nuraida, Nuri Andarwulan, Wisnu Ananta Kusuma

**Affiliations:** 1 Food Science Study Program, Graduate School, IPB University, Bogor, Indonesia; 2 Department of Food Science and Technology, IPB University, Bogor, Indonesia; 3 Department of Computer Science, Faculty of Mathematics and Natural Sciences, IPB University, Bogor, Indonesia

**Keywords:** Bacteria, excreta, civet coffee, NGS, pathogen.

## Abstract

**Objectives:**

Kopi luwak (civet coffee) is produced through a fermentation process in the digestive system of civets. This study aims to investigate the diversity of microorganisms in Indonesian civet excreta fed with robusta coffee cherries.

**Materials and Methods:**

Six excreta samples were collected from male and female Indonesian civets of three species: binturong (*Arctictis binturong*), Asian palm civet (*Paradoxurus hermaphroditus)*, and masked palm civet (*Paguma larvata).* Microbial diversity was analyzed using next-generation sequencing.

**Results:**

Based on alpha and beta diversity analysis, the microbial community in civet excreta differs. Microbes found in the excreta of male *P. hermaphroditus* (PH-M) were the most diverse. Microbes were identified in the bacterial and yeast domains. The relative abundance of bacteria was higher than yeast. The occurrence of non-pathogenic bacteria (50.76%–90.51%) was higher than pathogens (9.49%–41.24%). The dominant bacteria in the excreta of all civets were *Escherichia coli* (15.98%–54.68%). Although not dominant microorganisms, lactic acid bacteria (LAB) and yeast are present in civet excreta. The LAB present in the range from 0.16% to 32.14%, with the most abundant LAB being *Streptococcus pasteurianus*, *Weissella confusa*, and *W*. *cibaria.* Meanwhile, the identified yeasts were *Hanseniaspora opuntiae* and *H*. *uvarum*. Pathogenic bacteria, both spore-forming and non-spore-forming, were also present in civet excreta. The virulence factors and antimicrobial resistance gene cluster were detected.

**Conclusion:**

The microbial diversity of Indonesian civet excreta is influenced by species and sex. PH-M contains the most diverse microbes. The presence of foodborne pathogens in civet excreta may be carried over into the beans, and hence, further processing of the beans should assure the safety of the beans.

## Introduction

Kopi luwak (civet coffee) prepared from Arabica, Robusta, and Liberica coffee cherries, is a popular and highly valued coffee. Civet coffee is renowned for its unique flavor profile, which is attributed to its in vivo processing method—fermentation within the civet’s digestive system. The common civet species used for producing civet coffee is *Paradoxurus hermaphroditus* (PH), either in Indonesia [1, 2] or in Vietnam [3]. Other species, such as *Arctictis binturong* (AB) [4] and *Paguma larvata* (PL), are also used to produce Indonesian civet coffee. Coffee beans remain in the digestive system and mix with civet excreta for an extended period, approximately 12 h [5]. During the digestive process, biochemical reactions occur, but the exact mechanisms of which remain unclear. It is hypothesized that enzymatic reactions and microbial activity contribute to alterations in the chemical composition of the coffee beans. Studies in this area are still limited. Robusta civet coffee from Indonesia had lower protein [5], caffeine, sugar content, and pH [6], while fat and carbohydrate content was higher [5]. According to Yulia et al. [4], the caffeine content of civet coffee produced by *P*. *hermaphroditus* was lower than *A*. *binturong*. However, information on the presence of microbes in the digestive system or the excreta of A. binturong has not been comprehensively reported.

Previous research has studied the role of microbes in civet coffee fermentation by isolating and characterizing microbes from the digestive system and excreta of civets to use them as *in vitro* fermentation starters for civet coffee [7–10]. Previous researchers commonly isolated microbes from excreta and the digestive system of civets using selective media according to the targeted microbes [11–17]. Some microbes that have been isolated are known to be able to degrade cellulose [11, 16, 18, 19], protein [11, 12, 18, 19], pectin [20], lipids [16, 18], and caffeine [21].

Previous studies have not been able to describe all microorganisms in the civet’s digestive system and excreta because they only used methods to detect the presence of targeted culturable microbes. Non-targeted culturable microbes, as well as non-culturable microbes, were not identified. The only study detecting microbes in the excreta of civets using the next-generation sequencing (NGS) technique with the 16S rRNA gene metabarcoding approach was done by Watanabe et al. [22]. NGS techniques can provide more in-depth information on microbial diversity than selective media culture methods [23]. A study by Watanabe et al. [22] revealed that the microbiota in the excreta (feces) of *P. hermaphroditus* in Johor, Malaysia, was dominated by the genus *Gluconobacter*, followed by *Citrobacter*. The presence of other genera was not reported. In addition, species [24], feed [25], and sexes [26] affect the gut microbiome diversity of animals. Studies of microbial diversity in different civet species (Supplementary Fig. 2) and sexes that are used in Indonesian civet coffee production have not been conducted, so the topic is interesting to investigate.

Given the limited information regarding the abundance and diversity of microorganisms in civet excreta in Indonesia, this study aims to analyze the microbial profile of civet excreta fed with robusta coffee cherries. Sources of excreta were three species of domesticated civets commonly utilized for luwak coffee production in Indonesia: binturong (*A. binturong*), Asian palm civet (*P. hermaphroditus*), and masked palm civet (*P. larvata*); each from male and female civets. Analysis was conducted using the shotgun metagenomics approach. This approach is expected to provide deeper insights into microbial diversity, particularly those involved in *in vivo* fermentation, as well as the potential microbiological risks associated with civet coffee production.

## Materials and Methods

### Sample collection

**Supplementary Figure 1.**
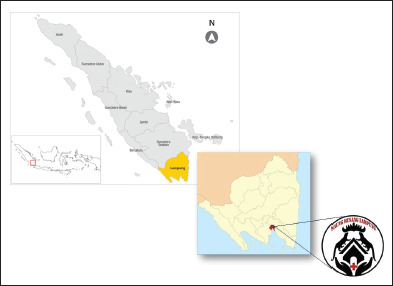
The location of sampling The map cited from https://id.wikipedia.org/wiki/Berkas:Peta_Wilayah_Sumatra.jpg, https://su.m.wikipedia.org/wiki/Gambar:Peta_ Lokasi_Kota_Bandarlampung.svg and icon of Saung Musang Lampung cited from https://www.instagram.com/saungmusanglampung/ Addres of Saung Musang Lampung : J6XX+6VW, Rajabasa Jaya, Rajabasa, Bandar Lampung ity, Lampung 35141

**Supplementary Figure 2.**
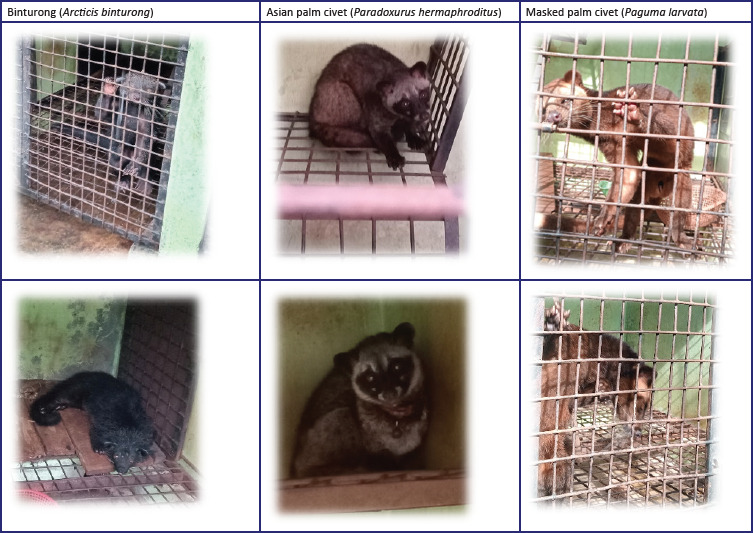
The picture of civets with different species

Fecal samples containing coffee beans were collected from civets at the Saung Musang Lampung, a civet farm at H. Sardana Street, No. 27, Rajabasa, Bandar Lampung City, Lampung Province, Indonesia (Supplementary Fig. 1). The sampling period was conducted in June 2024 (Supplementary Table 1). All civets are given the same feed: banana, papaya, boiled chicken head, and robusta coffee cherries. Coffee cherries are given during the coffee harvest period. The samples were obtained from three civet species: *P. hermaphroditus*, or Asian palm civet; *A. binturong* (Supplementary Fig. 2), or binturong; and *P. larvata* (PL, or Asian palm civet. Feces excreted by each male (M) and female (F) civet were immediately collected (1–5 gm), placed in vials, and preserved with 1–5 ml of RNA/DNA shield solution (Zymo Research, US). The samples were then stored in a 4°C refrigerator. The fecal collection was performed four times over 2 weeks. The collected excreta samples were subsequently homogenized for microbial profiling of each species and sex.

### DNA extraction and library preparation

DNA was extracted from excreta containing coffee beans using the ZymoBIOMICS DNA Miniprep Kit (Zymo Research D4300, US). The DNA extraction procedure follows the manufacturer’s protocol. DNA quality control included assessing DNA quantity, purity, and integrity to ensure suitability for library preparation and sequencing. DNA concentration was measured using the Qubit dsDNA Sensitivity to ensure sufficient quantity. DNA concentrations ranging from 0.1 to 120 ng/µl were deemed appropriate for library preparation. DNA purity was evaluated using a nanodrop spectrophotometer (Nanodrop 2000 Thermo Fisher Scientific, US), with acceptable A260/280 ratios ranging from 1.8 to 2.0. DNA integrity was assessed using agarose gel electrophoresis (1% TBE agarose). High-quality DNA was identified by clear bands >10 kb, indicating the absence of significant degradation [27].

Library preparation was conducted using xGEnTM DNA Library Prep EZ UNI (Integrated DNA Technologies, US). The sequence of the forward adapter was 5’-AGA TCG GAA GAG CAC ACG TCT GAA CTC CAG TCA-3’, and the reverse adapter was 5’-AGA TCG GAA GAG CGT GTA GGG AAA GAG TGT-3’. The quality and quantity of the prepared libraries were evaluated using a Tape Station (Agilent, US) and Qubit Fluorometer (Thermo Fisher Scientific, US).

### Sequencing and assembly of metagenomic data

Sequencing was performed using Illumina NextSeq 2000 (Illumina, US). The sample library size was 2 × 150 bp (paired-end) for 300 cycles. Raw sequencing data were subjected to quality control, including base content analysis, k-mer filtering, and adapter trimming, using fastp (v0.23.2) (https://github.com/OpenGene/fastp) [28]. Fastp is a tool designed to provide ultrafast all-in-one preprocessing and quality control for FastQC data. The cleaned reads were referred to as “reads.” Read quality was assessed using FastQC (v0.11.9), a quality control tool for high-throughput sequence data. (https://github.com/s-andrews/FastQC), and only reads with a Phred score > 30 were retained. FastQC analysis reports were compiled using MultiQC (v1.13) (https://github.com/ewels/MultiQC). It is a tool to create a single report with interactive plots for multiple bioinformatics analyses across many samples. The *de novo* assembly of reads was performed using Megahit (v1.2.9) (https://github.com/voutcn/megahit) [29]. Assembly quality was evaluated using Quast (v5.0.2) (https://github.com/ablab/quast), and assembly visualization was carried out using Bandage (0.8.1). Contigs data generated by assembly using Megahit in FASTA files are transformed into a SAM file using SAMtools (v1.6) [30]. Contigs with coverage less than 2x or a length shorter than 500 bp were filtered using BBTools (BBMap v37.62). Contigs have coverage > 2, and a minimum length of 500 bp is retained. This aims to eliminate contigs that come from noise or assembly errors. Short contigs are difficult to analyze further, such as for gene annotation.

### Taxonomic analysis and microbial virulence factors

Contigs larger than 500 bp were used in the alignment process. The number of contigs for each sample (Table 1) was aligned against the NCBI NR (Non-Redundant Protein Database) reference database using DIAMOND (v2.0.15) (https://github.com/bbuchfink/diamond) with BLASTx [31] because NCBI NR is a database containing protein sequences, so the contigs data cannot be directly aligned with NCBI NR. Therefore, we use DIAMOND. DIAMOND can align DNA sequences against the NCBI NR protein database. The DIAMOND alignment archive (DAA) file is used for further analysis with the MEGAN software. To visualize and analyze the data in MEGAN, the DAA file must first be converted into a format compatible with MEGAN. Taxonomic analysis was performed using MEGAN (V6.24.24) based on the lowest common ancestor (LCA) algorithm [32]. This algorithm works by finding the lowest taxonomy point still supported by the data, thus minimizing annotation errors. The LCA parameter was set as follows: minimum score: 50. This value was set to filter out the alignment errors. Top percent: 10%. This value was set to avoid bias from only one best hit. Minimum support percentage: 0.05%, and minimum support: 1. This value was set to keep all potentially relevant organisms under consideration, especially in low-coverage datasets.

**Table 1.** Quality of sequences from six samples.

	AB-F	AB-M	PH-F	PH-M	PL-F	PL-M
Reads (M)	49.632086	59.655666	49.921836	49.387318	70.821756	65.190578
Average length (bp)	140	136	140	141	135	138
Duplication rate (%)	9.88	12.92	12.87	13.31	10.11	10.39
GC content (%)	42.99	43.31	45.08	44.56	44.97	47.29
Contigs	33,586	105,541	247,423	103,338	158,440	92,540

*AB-F* = *Arctictis binturong*-female, *AB-M* = *Arctictis binturong*-male, *PH-F* = *Paradoxurus hermaproditus*-female, *PH-M* = *Paradoxurus hermaproditus*-male, *PL-F* = *Paguma larvata*-female, *PL-M* = *Paguma larvata*-male.

Microbial richness was analyzed using the Chao1 and ACE indices [33], while diversity was assessed using the Shannon and inverted Simpson indices [34]. The Simpson indices are less sensitive to richness compared to the Shannon index. However, the Simpson index is more sensitive to evenness. In this study, the Simpson’s index value was 0.99, making it challenging to interpret microbial diversity within the sample. Therefore, these indices were transformed into in reciprocal form (1/D), which is known as the reciprocal /inverted Simpson indices [35].

Beta diversity analysis was also conducted to evaluate variations in microbial community composition among samples using the Bray-Curtis dissimilarity [36]. Subsequently, non-metric multidimensional scaling (NMDS) was employed to reduce the dimensionality of the data and visualize the variations in community composition within a two-dimensional space. The NMDS analysis uses the metaMDS function from the vegan package in R.

Contig data were further analyzed to predict the presence of virulence factor genes and antimicrobial resistance. ORF detection was conducted using MetaGeneMark software [37]. Predicted ORFs shorter than 100 bp were filtered out using SeqKit. Filtered ORFs from each sample were combined. Redundant ORFs (100% identity) were removed using BBMap to create a unique gene catalog. The identified ORFs were compared against known protein databases to determine potential functions associated with the ORFs using the virulence factors (Supplementary Table 5) database (http://www.mgc.ac.cn/VFs/) [38]. Gene mapping was performed using DIAMOND BLAST. The same steps were applied to determine antibiotic resistance genes (ARGs) (Supplementary Table 6), and the ORFs were aligned against the comprehensive antibiotic resistance database [39]. Relative microbial abundance and prediction of Paradoxurus hermaproditus-male, PL-F = Paguma larvata-female, PL-M = Paguma larvata-male. virulence factor genes were visualized as heatmaps using the SRplot web-based tool [40].

## Results and Discussion

### Sequence quality

The sequencing results of the six quality-controlled samples are presented in Table 1. All purity values indicated that the DNA was of excellent quality, free from protein or phenol contaminations, and suitable for library preparations and sequencing processes. The total number of reads from the six samples ranged from 49 to 71 million, with the average read length ranging from 135 to 141 bp (Table 1). This low variation in read length among samples minimizes potential bias during mapping to the reference genome. Duplication rates ranged from 9.88% to 13.31%, with AB-F exhibiting the lowest duplication rate (9.88%), indicating broader diversity and reduced risk of analytical bias due to duplication. Similarly, GC content information in metagenomic data analysis ensures accurate taxonomic identification by reducing mapping bias. The GC content of civet feces DNA ranged from 43.31% to 47.39%, which is considered suitable for microbial mapping in samples [41]. Only reads with a Phred score of > 30 were carried forward to the assembly process. The number of contigs varied across the samples, as did the alpha diversity indices.

### Alpha diversity

**Figure 1.**
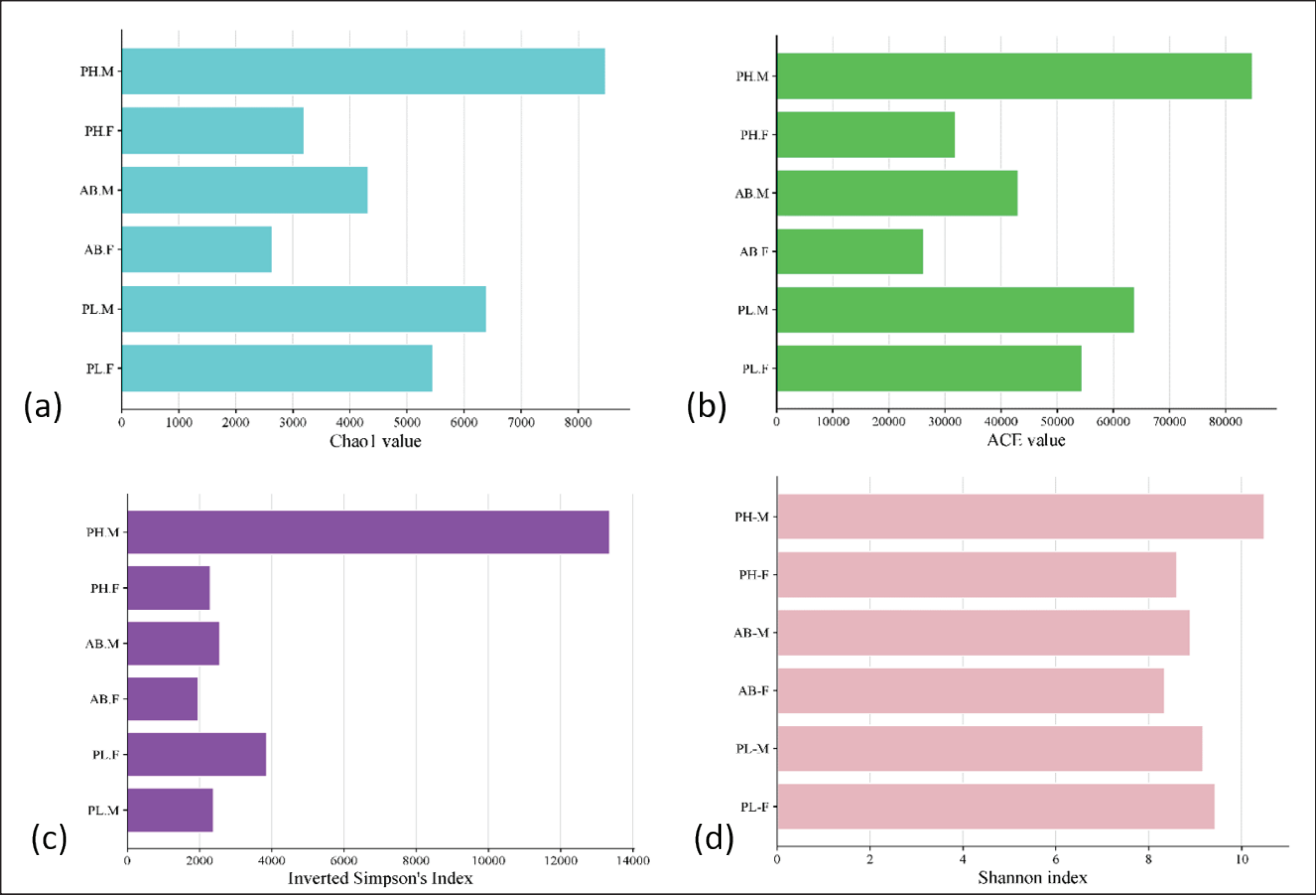
Indices for indicating microbial diversity using different approaches (a) Chao1, (b) ACE, (c) Inverted Simpson’s index, (d) Shannon index. *AB-F* = *Arctictis binturong-*female, *AB-M* = *Arctictis binturong-*male, *PH-F* = *Paradoxurus hermaproditus*female, *PH-M* = *Paradoxurus hermaproditus*-male, *PL-F* = *Paguma larvata*-female, *PL-M* = *Paguma larvata*-male.

Alpha diversity is a method used to measure the microbial community’s diversity. It is quantified using various indices to analyze the differences in community richness. In this study, the indices used were Chao1, abundance-based coverage estimator (ACE), Shannon, and inverted Simpson’s indices, as shown in Figure 1. The high value of each index indicated that the microbial diversity of excreta between the civet species and sexes was high. The overall test indicated that PH-M has high microbial diversity, while AB-F has the lowest microbial diversity. The Chao1 indices ranged from 26323.3 to 84784.7. The ACE indices value ranged from 26230.7 to 84773.4. The Shannon indices ranged from 8.3 to 10.4, indicating high microbial diversity within each civet species. According to Nolan et al. [42], the Shannon index value above 2 indicates high microbial diversity. The inverted Simpson’s indices value ranged from 1965.57 to 13363.14. These indices provide a holistic picture of alpha diversity; by combining these metrics, we achieved a robust and detailed understanding of the microbial community between groups.

### Beta diversity

**Figure 2.**
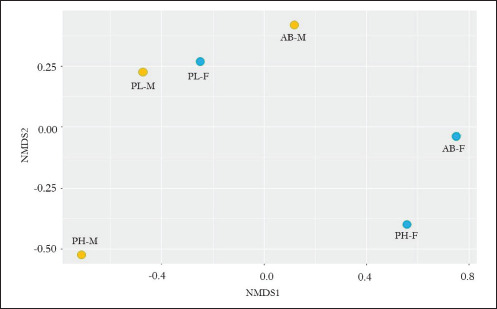
Non-metric Multidimensional Scaling (NMDS) plot based on Bray-Curtis dissimilarity value of different civet excreta. *AB-F* = *Arctictis binturong-*female, *AB-M* = *Arctictis binturong-*male, *PH-F* = *Paradoxurus hermaproditus*-female, *PH-M* = *Paradoxurus hermaproditus*-male, *PL-F* = *Paguma larvata*-female, *PL-M* = *Paguma larvata*-male.

Figure 2 shows the distance between samples in the NMDS plot. The distance between PL-M and PL-F is close (with the Bray-Curtis distance = 0.26), indicating no difference in the microbial community in the two civets’ excreta. The distance between AB-M and AB-F is spread further than PL-M and PL-F, indicating that the microbial community of both civet excreta is more diverse than PL-M and PL-F. The farthest distances are PH-M and PH-F (with the Bray-Curtis distance = 0.73), indicating that microbial communities in PH-M and PH-F differ.

**Figure 3.**
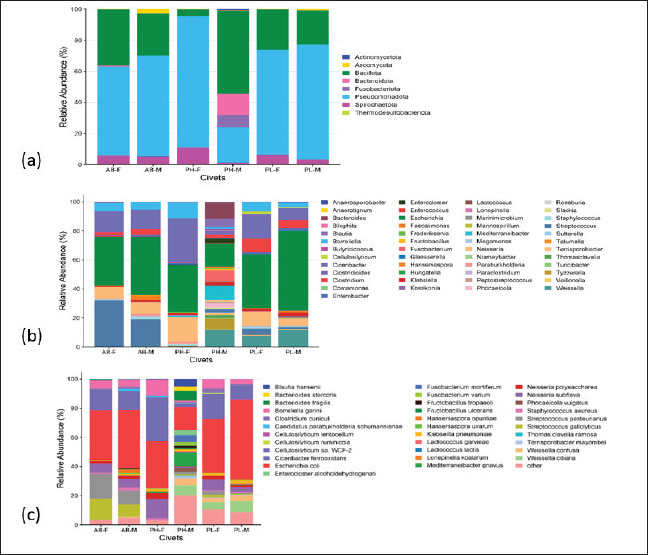
Microbial profile of civet excreta across different species and sexes. (a) At the phylum level, (b) at the genus level, and (c) at the species level. *AB-F* = *Arctictis binturong-*female, *AB-M* = *Arctictis binturong-*male, *PH-F* = *Paradoxurus hermaproditus*-female, *PH-M* = *Paradoxurus hermaproditus*-male, *PL-F* = *Paguma larvata*-female, *PL-M* = *Paguma larvata*-male.

Phylogenetic analysis of civets conducted by Patou et al. [43] demonstrated that *A. binturong* is closely related to *P. larvata*, while *P. hermap*hroditus share*s* a closer relationship with *P. larvata* and is more distantly related to *A. binturong*. These results suggest that genetic factors influence microbial communities, aligning with the patterns observed in Figure 3. This study is supported by Kovacs et al. [44], who reported that genetic factors affect rodent microbial composition. Similarly, differences in sex influence microbial composition due to variations in the hormonal system. Hormones such as testosterone [45] and estrogen [46] are known to affect the presence of microbes in the digestive system.

The limitation of the present study was its sample population. This study only took the excreta of one male or female civet per species. Hence, the study’s results still need to be validated in future studies to obtain more representative data by repeating more than once in each sample.

### Microbial taxonomy in civet excreta

Based on alpha and beta diversity analyses, microbial differences in excreta exist between different species and sexes. The shotgun metagenomics approach provided more comprehensive microbes present in civet excreta (Fig. 3), with a total of 8 phyla, 49 genera, 79 bacterial species, and 2 yeast species detected. The phylum Pseudomonadota was the dominant phylum in civet excreta (22.92%–84.82%), followed by Bacillota (3.92%–53.39%)*.* Pseudomonadota is commonly found in environmental habitats and enters the digestive system by ingestion. This phylum is prevalent in the oral cavity [47]. The abundance of Pseudomonadota is inversely correlated with the presence of Bacillota (Fig. 3a), formerly known as *Firmicutes* [48].

Phyla with relatively low abundance but present in all male and female civet species include *Spirochaetota* (1.09%–11.00%) and *Actinomycetota.* Bacteroidota and Fusobacteriota phyla exhibited a higher abundance in PH-M compared to the other five civet species. The relative abundance of these phyla was 13.41% and 7.99%. AB-M had the highest at 2.89% in the phylum *Ascomycota*.

At the genus level (Fig. 3b), genera identified in all civet excreta of civets with high relative abundance included *Escherichia, Ciceribacter, Streptococcus, Neisseria,* and *Borreliella* (Supplementary Table 2). Meanwhile, genera with low relative abundance included *Lactococcus, Weissella, Fructobacillus*, and *Hanseniaspora*. Based on alpha diversity analysis, the excreta of PH-M has a higher index value than the others. In the excreta of PH-M, the genera found to have a higher relative abundance compared to those in the other excreta were *Weissella, Bacteroides, Tyzzerella, Fusobacterium, Blautia, Fructobacillus,* and *Lactococcus,* while *Escherichia* was lower compared to other civet excreta. The relative abundance of *Streptococcus* was high in AB-F, followed by AB-M, while *Escherichia* was higher in PL-M. *Escherichia* is commonly found in the digestive system of mammals, including omnivores, carnivores, and herbivores [49].

Based on beta diversity analysis, there are differences in microbial communities in several species of civets. There are genera not found in AB-F excreta, namely *Anaerotignum, Marinimicrobium, Roseburia,* and *Veillonella. Anaerotignum* was also not found in AB-M, nor *Veillonella* in PH-F.

This study identified 79 species of bacteria and two species of yeast, species of microbes with high relative abundance, as shown in Figure 3c. Species of bacteria found in all the excreta of civets with high relative abundance included *Escherichia coli, Ciceribacter ferrooxidans*, and *Borreliella garinii.* Conversely, bacteria and yeast with low relative abundance yet found in all civets’ excreta were *Clostridium cuniculi, Neisseria polysaccharia, Neisseria subflava, Cellulosilyticum lentocellum, Cellulosilyticum ruminicola, Cellulosilyticum* sp. WCF-2, *Klebsiella pneumoniae, Hanseniaspora opuntiae, and Hanseniaspora uvarum*. In line with the alpha diversity analysis results, the civet excreta with high microbial diversity was PH-M. Bacteria found in high relative abundance in PH-M compared to others included Fructobacillus tropaeoli, Fructobacillus ulcerans, Lactococcus garvieae, Lactococcus lactis, Enterocloster alcoholdehydrogenati, and Fusobacterium mortiferum. In contrast, E. coli in PH-M was the lowest compared to other civet excreta.

Referring to the result of beta diversity analysis, it shows that microbial communities in the three species of civet are different. *Anaerotignum lactatifermentans* was not found in either *A. binturong* or *Blautia* sp. Marseille-P3201T was not found in AB-F and PL-F. *Marinimicrobium alkaliphilum, Roseburia hominis,* and *Slackia piriformis* were not found in AB-F. *Veillonella ratti* was not found in AB-F and PH-F.

Comparing the present results to the previous studies on the excreta of *P. hermaphroditus* [16, 17, 18, 21], one similar species was found, namely *W*. *cibaria.* The present research revealed that *E*. *coli* was the dominant species in the civet excreta. *E*. *coli* has been found in the stomach, small intestine, and large intestine of *P. hermaphroditus* [50]. Watanabe et al. [22], using a metabarcoding approach using 16S rRNA, also reported the presence of *Escherichia* in wild civet excreta, although it was not the dominant bacterium. Environmental factors such as habitat and dietary intake may influence microbial diversity.

### Presence of lactic acid bacteria (LAB) and yeasts

Previous studies commonly targeted LAB to be isolated from civet coffee and studied for their physiological properties [14, 16–21] or applied in coffee fermentation to produce coffee with similar quality to civet coffee [9, 10]. In addition to LAB (Supplementary Table 3), yeast in excreta has been isolated [12]. The use of a combination of LAB and yeast improved the sensory quality of coffee brewing compared to the control (the coffee without fermentation) [51].

**Figure 4.**
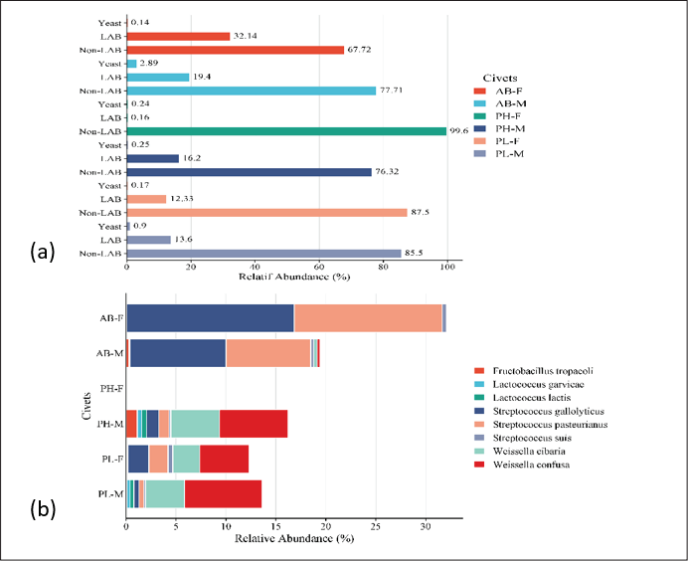
Relative abundance of LAB, non-LAB, and yeast (a), and LAB species in civet excreta (b). AB-F = *Arctictis binturong-*female, AB-M = *Arctictis binturong-*male, PH-F = *Paradoxurus hermaproditus*-female, PH-M = *Paradoxurus hermaproditus*-male, PL-F = *Paguma larvata*-female, PL-M = *Paguma larvata*-male.

In this study, the relative abundance of LAB in the civet excreta was lower than other bacteria (0.16%–32.1%). The abundance of LAB was higher in the excreta of binturongs (32.1%) compared to other civets (Fig. 4a; Supplementary Table 2). *Streptococcus gallolyticus* and *S. pasteurianus* were the dominant LAB in the excreta of both male and female binturongs. *Weisella cibaria* and *W*. *confuse* were predominantly found in the excreta of male Asian palm civets (PH-M), as well as female and male masked palm civets. *F*. *tropaeoli, L*. *garvieae,* and *L*. *lactis* exhibited higher relative abundance in the excreta PH-M. *Streptococcus suis* was detected in all civets’ excreta, with a relative abundance ranging from 0.02% to 0.46%, as shown in Figure 4b and Supplementary Table 3.

Previous studies suggested that LAB isolated from civet excreta may contribute to the fermentation process of civet coffee. *S*. *gallolyticus* produces tannase, which can convert gallotannins through decarboxylation, forming acid. The formation of acids is a result of the breakdown of starch, glycogen, inulin, lactose, mannitol, raffinose, trehalose, and methyl-β-D-glucopyranoside. This bacterium is also capable of hydrolyzing esculin and producing β-glucosidase and α-galactosidase [52]. Similarly, *S*. *pasteurianus* can produce acetoin and acids from lactose and trehalose. In addition to β-glucosidase, this bacterium produces β-glucuronidase and β-mannosidase [53]. Likewise, *S*. *suis* shares similar characteristics in producing enzymes and acids with the two previously mentioned species [54].

Several LAB identified in civet excreta are listed by EFSA as safe to be used in the production of fermented food, including *W*. *cibaria* and *W*. *confusa* [55]. *W*. *cibaria* isolated from the civet digestive system has proteolytic and lipolytic properties [18, 19]. *L*. *lactis,* which is also commonly used for food fermentation, is proteolytic and capable of hydrolyzing pectin [20]. *F*. *tropaeoli* is the most commonly found in PH-M (1.11%). This bacterium produces acids from the metabolism of fructose, glucose, and mannitol. It grows well at pH 4–8, a temperature of 10°C–15°C, and a low salt concentration (2.5%) [56].

In addition to LAB, this study has also successfully identified the presence of yeast (Fig. 4a). The relative abundance of yeast is much lower compared to bacteria, ranging from 0.24 to 2.89%. The present study also detected yeast in civet excreta (Fig. 3c). The yeasts identified in this study were *H*. *opuntiae* and *H*. *uvarum. H*. *opuntiae* was commonly used in ethanol production from various carbon sources [57]. Although their presence is relatively small, it is suspected that both yeasts contribute to the fermentation of coffee cherries in the civet’s digestive system. *H*. *opuntiae* has been used in wet coffee fermentation, and its presence can inhibit the growth of the mycotoxin-producing mold *Aspergillus ochraceus* [58] while producing a fruity profile and achieving a high cupping test score [59]. Similarly, *H*. *uvarum* has been utilized in coffee fermentation. This yeast is inoculated into post-harvest coffee processing as a single culture [60] or in combination with other species. Coffee fermented with mixed yeast cultures shows improved aroma and sensory scores with a roasted almond aroma [61]. This yeast can produce pectinase, chitinase, protease, and β-glucosidase [62]. The activity of these enzymes is beneficial in the production of terpenes and esters, enhancing floral and fruity notes [63].

### Presence of pathogenic microbes

**Figure 5.**
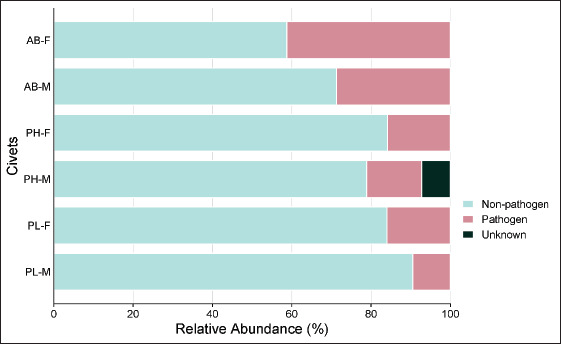
Relative abundance of non-pathogenic and pathogenic microbes in civet excreta. *AB-F* = *Arctictis binturong-*Female, *AB-M* = *Arctictis binturong-*Male, *PH-F* = *Paradoxurus hermaproditus*-Female, *PH-M* = *Paradoxurus hermaproditus*-Male, *PL-F* = *Paguma larvata*-Female, *PL-M* = *Paguma larvata*-Male.

**Figure 6.**
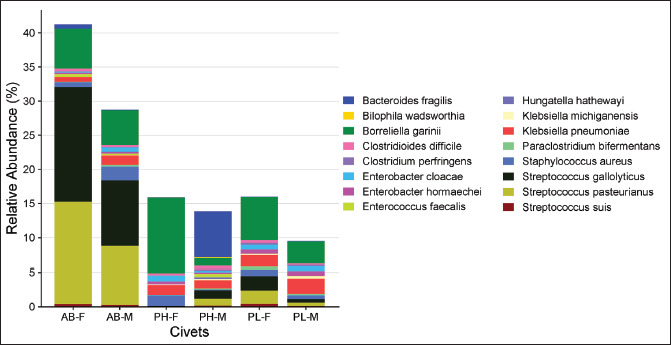
Relative abundance of pathogenic bacteria in civet excreta. *AB-F* = *Arctictis binturong-*female, *AB-M* = *Arctictis binturong-*male, *PH-F* = *Paradoxurus hermaproditus*-female, *PH-M* = *Paradoxurus hermaproditus*-male, *PL-F* = *Paguma larvata*-female, *PL-M* = *Paguma larvata*-male.

The present research detected the presence of pathogenic bacteria in civet excreta, with their relative abundance lower than non-pathogenic bacteria. The relative abundance of non-pathogenic microbes ranged from 58.76% to 90.51%, while pathogenic bacteria ranged from 9.49% to 41.24% (Fig. 5; Supplementary Table 4). Excreta of PH-M carried high pathogenic bacteria, followed by PH-F, while PL-M was the lowest (Fig. 6). Pathogens with high relative abundance were *S*. *gallolyticus* and *S*. *pasteurianus*, which were found in excreta of AB-F and AB-M, while *Borellia garinii* was found in excreta of PH-F.

Some pathogenic bacteria are known as food-borne pathogens, including *Clostridium difficile, C*. *perfringens, Enterobacter cloacae, E*. *faecalis, K. pneumoniae, Paraclostridium bifermentans,* and *S*. *aureus*. Those bacteria have different pathogenies through infection or toxin formation. Bacteria that can infect humans are *C*. *difficile* [64, 65], *C*. *perfringes* [66], *E*. *cloacae* [67], *E*. *faecalis* [68], and *K*. *pneumoniae* [69], while *S*. *aureus* causes illness in the host due to the toxin it produces in foods [70].

The pathogenic bacteria have low relative abundance but are still a concern. Some bacteria produce spores that are heat resistant, including *C*. *perfringens, C*. *difficile,* and *P*. *bifermentans. C*. *perfringens* can produce endospores and lethal toxins in the gastrointestinal tract. *C. perfringens* is a foodborne pathogen that causes infection and produces toxins when ingested. This bacterium also secretes sialidases (*neuraminidases*) [71, 72], enzymes capable of hydrolyzing oligosaccharides containing sialic acid. Sialylated glycans help protect the host epithelial cell from bacterial attack [73]. Likewise, *P*. *bifermentans* is a spore former [74], causing infection and potentially producing toxins [75], but it is considered a rare human pathogen [76], although it is potentially a foodborne pathogen [77]. Similar to *C*. *perfringens*, which produce heat-resistant spores, the spores of *C*. *difficile* could germinate in foods and cause infection when they are ingested [78].

The coffee roasting process is generally carried out at 250°C for 3 to 8 min [79]. Although the survival of those spore-forming bacteria during coffee roasting has not been evaluated, their survival in the roasting process of beef has been reported [80]. Therefore, the survival of those spore-forming bacteria during the roasting process of civet coffee needs to be further studied.

In addition to foodborne pathogens, pathogenic bacteria that can infect humans directly were also found in civet excreta, including *Bacteroides fragilis, Bilophila wadsworthia, Borreliella garinii, Enterobacter hormaechei, Hungatella hathaway, K. michiganensis, S*. *gallolyticus*, *S*. *pasteurianus,* and *S*. *suis.* The possibility of transferring pathogenic bacteria from excreta to humans should be considered in civet coffee production to prevent worker infection.

### Virulence factors and antimicrobial resistance gene clusters in civet excreta

**Figure 7.**
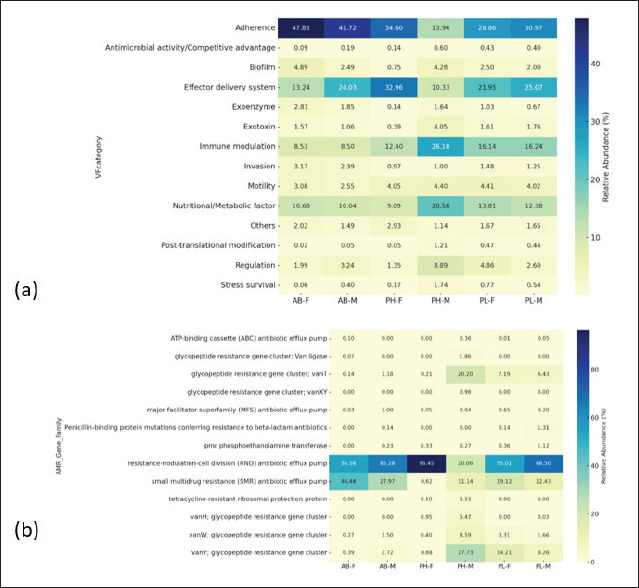
Microbial virulence factors (a) and antimicrobial resistance gene clusters in civet excreta (b). *AB-F* = *Arctictis binturong-*female, *AB-M* = *Arctictis binturong-*male, *PH-F* = *Paradoxurus hermaproditus*-female, *PH-M* = *Paradoxurus hermaproditus*-male, *PL-F* = *Paguma larvata*-female, *PL-M* = *Paguma larvata*-male.

Virulence factors and antimicrobial-resistant genes in civet excreta are shown in Figure 7a and 7b, and Supplementary Table 5 and 6. Virulence factors with high relative abundance in all civet excreta were adherence, effector delivery system, immune modulation, and metabolic factors, in contrast to biofilm, exoenzyme, exotoxin, invasion, motility, post-translational modification, regulation, and stress survival (Fig. 7a). Each microbe exhibits different virulence factors, but this study is unable to identify them in individual microorganisms present in civet excreta. Adherence is an important virulence factor in pathogenic bacteria. An infection starts when bacteria attach themselves to the host. The adhesion mechanism to the host can occur through biofilm formation or the presence of pili and fimbriae. Based on studies by Heilmann et al. [81], *S*. *aureus* can attach to the host because it can form fibronectin-binding proteins (FnBPs); *C*. *difficile* through the formation of biofilm and flagella [82]; and *K*. *pneumoniae* through the formation of polysaccharide capsule (CPS) and fimbriae [83].

Figure 7b shows that antimicrobial-resistant genes with high abundance (20.1%–96.4%) are genes encoding the resistance-nodulation-cell division (RND) antibiotic efflux pump. That is found in all excreta of civets. RND pumps function to expel antibiotics that enter bacterial cells. A small multidrug-resistant (SMR) antibiotic efflux pump was found with the second-highest abundance after the RND antibiotic efflux pump, which ranged from 0.62% to 44.44%. Microbial pathogens with SMR antibiotic efflux pumps can withstand antibiotic treatment, so preventing infection will be more difficult. Based on studies by Fernando et al. [84], the RND antibiotic efflux pump was found in Gram-negative bacteria, such as E. coli [85]. That is also found in S. aureus [86], K. pneumoniae [87], C. difficile [88], C. perfringens [89], and B. fragilis [90].

Results of the studies also found antibiotic-resistant gene clusters in the glycopeptide antibiotic class, including *van*T, *van*XY, *van*H, *van*W, and *van*Y. The relative abundance of the clusters’ genes in each civet excreta was low, except for *van*T (20.20%) and *van*Y (27.73%) in PH-M excreta (Fig. 7b). *van*T and *van*XY were found in *E*. *cassiflavus* [91], *van*H was found in *E*. *faecium* BM4147 [92], *van*Y was found in Bacillaceae [93], *Nonomuraea gerenzanensis*, and *N. gerenzanensis* pST30 [94].

In addition, penicillin-binding protein mutations conferring resistance to beta-lactam antibiotics were found in AB-M, PL-F, and PL-M excreta. Lim et al. [95] found it in *S*. *aureus.* Genes encoding tetracycline-resistant ribosomal protection protein were also found in PH-M and PH-F excreta. *Tet* genes encode tetracycline-resistant diversity in microbes, such as the *tet*(S) gene owned by *Listeria monocytogenes* [96], *tet*(Q) owned by *Bacteroides* [97], and *tet*(M), *tet*(O), and *tet*(S) found in *Streptococcus pyrogenes* [98] and *L. lactis* [99].

The present research is the first research report on virulence factors and ARG clusters present in the excreta of civets fed with coffee cherries as applied in civet coffee production.

## Conclusion

Based on the beta diversity analysis, species and sexes affect the diversity and abundance of microbes in civet excreta. Based on alpha diversity, PH-M has the highest microbial diversity compared to the other civets. Bacteria are the dominant microorganism, followed by yeast. The phylum Pseudomonadota was the dominant phylum in civet excreta. At the genus level, *Escherichia*, *Ciceribacter, Streptococcus,* and *Neisseria* have a high relative abundance, while at the species level, *Escherichia coli, Ciceribacter ferrooxidans, Neisseria subflava,* and *Borreliella garinii* also have high relative abundance. Although not dominant microorganisms, LAB and yeast are present in civet excreta, with the relative abundance of LAB ranging from 0.16% to 32.1% and yeast ranging from 0.24% to 2.89%. Pathogenic bacteria were also detected in civets’ excreta, ranging from 9.14% to 40.83%, with their identified virulence factors and antimicrobial resistance gene cluster. Pathogenic microbes raise concerns, highlighting the need for studies on microbial contamination of coffee beans and their survival during further processing. Future research should address the role of microorganisms in civet coffee during *in vivo* fermentation and the fate of pathogenic bacteria present in civet excreta during civet coffee processing to ensure the safety of civet coffee.

## Figures and Tables

**Supplementary Table 1.** Schedule of sampling

Sampling period	Time
I	16 June 2024
II	18 June 2024
III	24 June2024
IV	26 June 2024

**Supplementary Table 2.** Relative abundance of microorganisms (non LAB, LAB, and yeast)

Group of Microbes	AB-F	AB-M	PH-F	PH-M	PL-F	PL-M
Non LAB	67.72176	77.70629	99.59885	76.32386	87.49852	85.50344
LAB	32.13632	19.3988	0.160912	16.20288	12.33463	13.60057
Yeast	0.141919	2.894919	0.240238	0.249668	0.166848	0.895987

**Supplementary Table 3.** Relative abundance of Lactic acid bacteria

LAB	Relative Abundance (%)					
	AB-F	AB-M	PH-F	PH-M	PL-F	PL-M
Fructobacillus tropaeoli	0.022339	0.284799	0.000301	1.105905	0.007302	0.075506
Lactococcus garvieae	0.00013	0.083703	0.000938	0.456482	0.061936	0.305058
Lactococcus lactis	0.00024	0.015113	0.000151	0.482964	0.096825	0.380799
Streptococcus gallolyticus	16.8097	9.610839	0.071914	1.225226	2.100479	0.525695
Streptococcus pasteurianus	14.82948	8.495098	0.066401	1.024865	1.93921	0.48263
Streptococcus suis	0.442676	0.299644	0.015752	0.1706	0.455807	0.150309
Weissella cibaria	0.026292	0.363947	0.00256	4.876461	2.71453	3.924688
Weissella confusa	0.00547	0.245653	0.002896	6.860378	4.95854	7.75589

**Supplementary Table 4.** Relative abundance of pathogens

Microbes	AB-M	AB-F	PH-M	PH-F	PL-M	PL-F
Hanseniaspora opuntiae	0.111585	2.572745	0.161305	0.20883	0.123436	0.7715
Clostridium cuniculi	0.730498	1.511863	0.193944	0.551614	2.455262	1.508423
Staphylococcus aeureus	0.727164	2.034341	1.525225	0.142238	0.917383	0.514325
Streptococcus pasteurianus	16.8097	9.610839	0.071914	1.225226	2.100479	0.525695
Streptococcus gallolyticus	14.82948	8.495098	0.066401	1.024865	1.93921	0.48263
Candidatus parabukholderia schumannianae	0.102052	1.443461	1.356079	0.019801	0.22403	0.318706
Neisseria polysaccharea	1.724162	1.945504	3.907337	0.297213	2.360596	1.140485
Neisseria subflava	6.394649	6.011279	13.24138	1.078694	7.4325	3.681765
Ciceribacter ferrooxidans	13.99436	13.06368	29.71213	2.428305	16.6417	8.332768
Klebseilla pneumoniae	0.655895	1.426288	1.475804	1.237709	1.649617	2.239012
Borreliella garinii	5.841834	5.115788	11.00257	1.094286	6.395404	3.219913
Fusobacterium mortiferum	6.99E-05	0.000947	0.000162	4.994468	0.00073	0.000645
Fusobacterium varium	0.022169	0.000924	0.000324	2.541324	0.000952	0.000855
Phocaeicola vulgatus	0.001168	0.012651	0.001054	2.6818	0.047214	0.013322
Bacterioides stercoris	0.000309	0.000813	2.32E-05	3.033096	0.000302	0.000436
Bacterioides fragilis	0.625351	0.006682	0.002166	6.710433	0.001238	0.003628
Thomas clavelia ramosa	0.002735	0.003296	0.004726	0.447759	0.013167	0.014939
Microbes	AB-M	AB-F	PH-M	PH-F	PL-M	PL-F
Enterocloster alcoholdehydrogenati	0.000429	0.000601	0.238118	3.196413	0.013825	0.006418
Blautia hansenii	0.000619	0.001492	0.102862	5.010754	0.001778	0.002899
Mediterraneibacter gnavus	0.033379	0.02459	0.808218	9.103235	0.024492	0.093243
Terrisporobacter mayombei	0.000978	0.002617	0.003289	1.329386	0.404402	0.558773
Fructobacillus tropaeolii	0.022339	0.284799	0.000301	1.105905	0.007302	0.075506
Weissella confusa	0.026292	0.363947	0.00256	4.876461	2.71453	3.924688
Weissella cibaria	0.00547	0.245653	0.002896	6.860378	4.95854	7.75589
Lonepinella koalarum	0.000559	0.013286	0.375229	0.284179	0.039531	0.191044
other	3.385219	5.674984	3.138383	23.31022	12.41086	9.943911

**Supplementary Table 5.** Relative abundance of virulence factors

Sampel	AB-F	AB-M	PH-F	PH-M	PL-F	PL-M
Adherence	47.83	41.72	34.60	13.96	28.86	30.97
Antimicrobial activity	0.09	0.19	0.14	0.60	0.43	0.40
Biofilm	4.89	2.49	0.75	4.28	2.50	2.00
Effector delivery system	13.24	24.03	32.96	10.33	21.95	25.07
Exoenzyme	2.81	1.85	0.14	1.64	1.03	0.67
Exotoxin	1.57	1.06	0.39	4.05	1.61	1.76
Immune modulation	8.53	8.50	12.40	26.18	16.14	16.24
Invasion	3.17	2.39	0.97	1.00	1.48	1.25
Motility	3.08	2.55	4.05	4.40	4.41	4.02
Nutritional/Metabolic factor	10.68	10.04	9.09	20.58	13.81	12.38
Others	2.02	1.49	2.93	1.14	1.67	1.65
Post-translational modification	0.02	0.05	0.05	1.21	0.47	0.44
Regulation	1.99	3.24	1.35	8.89	4.86	2.60
Stress survival	0.06	0.40	0.17	1.74	0.77	0.54

**Supplementary Table 6.** Relative abundance of ARG

AMR_Gene_Family			AB-F	AB-M	PH-F	PH-M	PL-F	PL-M
ATP-binding cassette (ABC) antibiotic efflux pump			0.10044483	0	0	0.3589445	0.0124797	0.0530263
glycopeptide resistance gene cluster; Van ligase			0.07174631	0	0	2	0	0
glycopeptide resistance gene cluster; vanT			0.14349261	1	0.214388	20	7	6
glycopeptide resistance gene cluster; vanXY			0	0	0	0.9604191	0	0
major facilitator superfamily (MFS) antibiotic efflux pump			0.02869852	0.99728	0.047642	0.0388048	0.6489455	0.0452996
Penicillin-binding protein mutations conferring resistance to beta-lactam antibiotics			0	0.135993	0	0	0.1372769	1
pmr phosphoethanolamine transferase			0	0.226655	0.333492	0.2716337	0.3619119	1
resistance-nodulation-cell division (RND) antibiotic efflux pump			69	55	65	96	20	55
small multidrug resistance (SMR) antibiotic efflux pump			44	28	0.619343	11	19	12
tetracycline-resistant ribosomal protection protein			0	0	0.095283	3	0	0
vanH; glycopeptide resistance gene cluster			0	0	0.952835	5	0	0.0303007
vanW; glycopeptide resistance gene cluster			0.27263596	1	0.404955	9	3	2
vanY; glycopeptide resistance gene cluster			0.38743005	3	0.881372	28	14	8

## List of abbreviations

µl, microliter

ABF, *Arctictis binturong-*female

ACE, abundance-based coverage estimator

ARGs, antibiotic resistance genes

CARD, comprehensive antibiotic resistance database

CPS, polysaccharide capsule

DNA, deoxyribonucleic acid

FnBPs, fibronectin-binding proteins

h, hours

LAB, lactic acid bacteria

LCA, lowest common ancestor

min, minutes

ml, milliliter

NGS, next generation sequencing

NMDS, Non-metric Multidimensional Scaling

ORF, Open Reading Frame

PH-M, *Paradoxurus hermaproditus* male

RNA, ribonucleic acid

RND, resistance-nodulation-cell division

rRNA, ribosomal RNA

SMR, small multidrug-resistant

VFDB, Virulence Factors Database.
